# An Assessment of the Effectiveness of Warfarin Therapy Monitoring Systems on Thrombophilic Patients in Zimbabwe

**DOI:** 10.1055/s-0038-1672186

**Published:** 2018-09-26

**Authors:** Aaron Maramba, Silenkosi Ncube, Arthur Mandisodza, Dayana Da Silva Marques, Tsehay Matsikure

**Affiliations:** 1 Department of Medical Laboratory Sciences, College of Health Sciences, University of Zimbabwe, Harare, Zimbabwe; 2 Department of Haematology, College of Health Sciences, University of Zimbabwe, Harare, Zimbabwe; 3 Department of Haematology, National Public Health Laboratories, Parirenyatwa Group of Hospitals, Harare, Zimbabwe

**Keywords:** anticoagulation, assessment, effectiveness, monitoring, thrombosis, warfarin

## Abstract

**Introduction** Thrombophilia describes conditions that predispose individuals to increased blood clotting and includes conditions such as deep vein thrombosis and pulmonary embolism. Thrombophilia is associated with high morbidity and mortality rates, and is commonly treated by warfarin anticoagulation. However, warfarin may cause both bleeding and clotting episodes if the therapy is not monitored and managed effectively.

**Objectives** The main objective of this study was to assess the effectiveness of warfarin monitoring systems on thrombophilic patients at a major hospital in Zimbabwe.

**Material and Methods** A clinical and laboratory prospective and retrospective study was performed on patients who had been on warfarin therapy for at least 1 year. Questionnaires were administered to participants on warfarin from outpatients clinics at Parirenyatwa Group of Hospitals. Their international normalized ratio (INR) results were also accessed from the laboratory information system and captured in the *Epi info* and Microsoft Excel for analysis.

**Results** Fifty questionnaires were administered and 47 (94%) participants responded adequately. Twenty-nine (61.1%) participants on warfarin were females. The majority of them were elderly and in the 31 to 40 age groups. Eighteen (38.3%) participants missed their medication at some point, while 12 (25.5%) had warfarin overdose. Sixteen (34%) and 11 (23.4%) admitted to taking alcohol and smoking, respectively, while on warfarin. Thirty-five (74.5%) did not change their medication nor were advised on the right diet. Thirty-four (72.3%) had appointments set after every month. Some participants indicated that they had symptoms of both clotting and bleeding. There were statistically significant differences ( *p* < 0.0001) between INRs for 3 monthly intervals from the initiation of warfarin therapy.

**Conclusion** Women and the elderly formed the majority of the patients on warfarin, indicating gender and advanced age susceptibility to thrombophilia, respectively. The effectiveness of the warfarin monitoring systems appeared to be hampered by lack of a coordinated system that adequately monitors anticoagulant therapy in the country.

## Introduction

Thrombophilia describes conditions of the hemostatic processes that tend to predispose individuals to clotting. Deep vein thrombosis (DVT) and pulmonary embolism (PE) are common examples and are collectively referred to as venous thromboembolism (VTE). 1

There are genetic and acquired forms of thrombophilia. The genetic forms are associated with deficiencies or abnormalities in mechanisms that control clotting, such as mutant factor V Leiden, protein C and S deficiencies, defective fibrinogen, and prothrombin allele G20210A mutation. 1 2 The acquired forms are associated with conditions that render individuals susceptible to clotting such as lengthy hospitalization, pregnancy, malignancy, inflammation, and immobility. Other blood disorders such as myeloproliferative disease and blood viscosity are now known to cause clotting. 1 Thrombophilia is a major cause of mortality; therefore, early diagnosis and treatment are vital.

Diagnosis of thrombophilia includes clinical findings and specific laboratory tests. Laboratory diagnosis includes tests that identify both genetic and acquired forms of thrombophilia. The major treatment formulae for thrombophilia are anticoagulation using heparin and/or warfarin. Heparin is given intravenously because it is not absorbed through the gut, while warfarin is given orally. 3 4 5

Warfarin prevents blood clotting by inhibiting vitamin K–dependent coagulation factors (II, VII, IX, and X). 1 6 7 However, despite its effectiveness, warfarin has several contraindications. Therefore, warfarin activity should be carefully monitored to avoid contraindications that may lead to bleeding or thrombosis. Careful monitoring of warfarin is usually done using prothrombin time, activated partial thromboplastin time, and international normalized ratio (INR). Monitoring of warfarin administration ensures that patients are taking adequate and safe doses of the drug. A high INR predisposes patients to an increased risk of bleeding, while an INR below therapeutic target indicates that the dose of warfarin is insufficient to protect against thromboembolic events. 1 8 9

The absence of a centralized coagulation clinic in Zimbabwe makes it difficult to monitor warfarin therapy in patients with thrombophilia. Patients are usually managed under individual specialized systems. There is also no standardized approach for the management of warfarin therapy. The increasing burden of thrombophilia now requires effective systems in place for monitoring warfarin therapy. The study aimed at assessing the effectiveness and identifying gaps in warfarin monitoring systems in the country.

## Materials and Methods

The present study was performed after approval from the local Joint Research Ethics Committee of the Parirenyatwa Group of Hospitals and University of Zimbabwe College of Health Sciences ( *JREC/387/16* ). Access to patients visiting various outpatient clinics of the Parirenyatwa Group of Hospitals and laboratory test results was granted by the clinical director and chief medical laboratory scientist, respectively, and each participant was required to give a written consent.

The study had a mixture of prospective and retrospective components from a cross-section of clinical and laboratory datasets. The study focused on outpatients who had been on warfarin therapy (following an event) for at least 1 year. The *inclusion criteria* were: patients on warfarin for at least 1 year; over 18 years of age; and a confirmed diagnosis of thrombophilia. The *exclusion criteria* were: patients with chronic disorders or malignancies involving the bone marrow as well as those below the age of 18 years.

Demographic and laboratory data were collected from 47 adult patients on warfarin who attended various outpatient clinics from January to April 2017. Questionnaires were administered to assess participants' knowledge of warfarin therapy, as well as its effects and recommendations on centralized warfarin clinic for ease of monitoring.

Coagulation test results performed by qualified and registered medical laboratory scientists on the STA-R Max (Stago) analyzer were accessed from the hematology laboratory information system. Data were captured on the Epi info and Microsoft Excel. Statistical analysis was done using the Graft Paired Prism software. Responses from participants were also captured into the Epi info for analysis.

## Results

A total of 50 questionnaires were administered to patients on warfarin and 47 participants fully and legibly answered the questionnaires, giving a return rate of 94%. The demographic distribution of participants showed that more female (61.7%) participants than males (38.3%) were on warfarin and that the majority (34%) of them were elderly (>50 years of age; Table 1 ). The age range of patients on warfarin was 24 to 71 years, with a median of 44 years.

**Table 1:** Demographic distribution of patients on warfarin therapy

Variable		Frequency	Percentage
Gender	Female	29	61.70%
	Male	18	38.30%
Age (y)	20–30	8	17.02%
	31–40	15	32.92%
	41–50	8	17.02%
	>50	16	34.04%

Twenty-nine (61.7%) of the 47 participants on warfarin indicated that they were not aware of the need for regular check-ups. Eighteen (38.3%) of the 47 had at some point missed their warfarin medication, while 12 (25.5%) had taken an overdose of warfarin at some point. Sixteen (34.0%) and 11 (23.4%) admitted taking alcohol and smoking, respectively.

Assessment of the warfarin administration systems at Parirenyatwa Group of Hospitals indicated that 35 (74.5%) of the participants were not asked to change their medication (if they were taking any contraindicated medicines) or their diet, according to the dictates of warfarin management. These warfarin patients were being managed by physicians and nurses under different clinics—some as medical patients and others as postsurgical patients.

Questionnaire responses on appointment frequency indicated that 4 (8.5%), 9 (19.2%), and 34 (72.3%) had appointments set at once in 3 days, once a week, and once monthly, respectively. Responses on the effects of warfarin indicated that 24 (51.1%) and 20 (42.6%) had experienced symptoms of clotting (chest pain, shortness of breath, and painful arms or legs) and unusual bleeding episodes, respectively. Twenty-one (44.7%) were on other long-term medication besides warfarin.

**Fig. 1:**
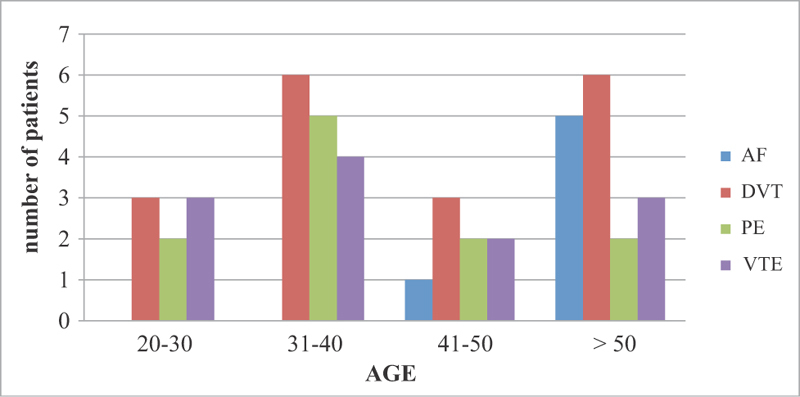
Distribution of warfarin indications by age groups of patients.

**Fig. 2:**
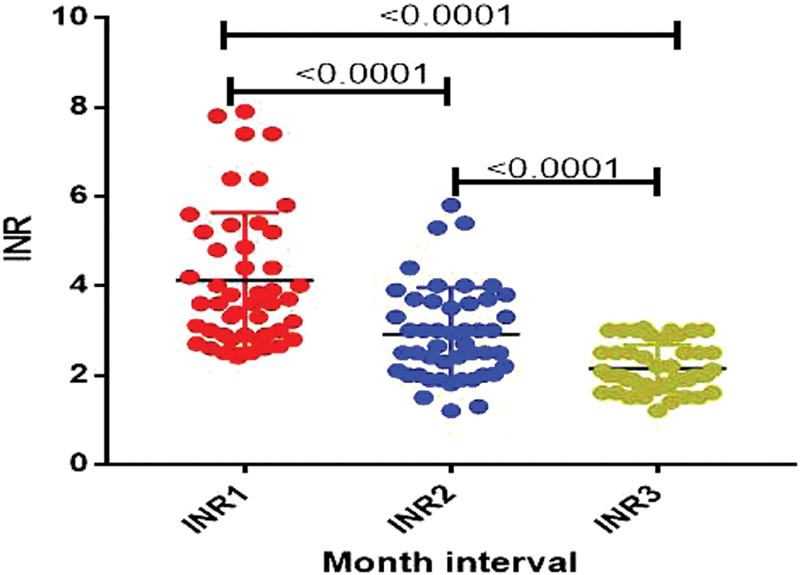
Effects of warfarin on INR results on three 1-month intervals.

DVT was the most common clinical indication for warfarin administration in all age groups, peaking in young adults (31–40-year-olds) and the elderly. Although PE and VTE were the most common conditions in all age groups, they were found to be highest in young adults. Atrial fibrillation was only found in the elderly age group ( Fig. 1 ). There were statistically significant differences ( *p* < 0.001) between patients' INR results for the three groups when divided into monthly intervals; one-month intervals from the initiation of warfarin therapy demonstrated a steady decline ( Fig. 2 ) indicating stabilization of the INR due to warfarin therapy.

## Discussion

The present study was performed over 4 months across different outpatient clinics where 50 questionnaires were administered and 47 responses were received. The responses to the administered questionnaires were good (94%), considering the duration over which the study was performed. This could be attributed to the country's high literacy rate.

There were more female participants than males, which could be a reflection of the country's demographic distribution as revealed by the last census figures. Other studies have shown a higher risk of VTE in women of childbearing ages. The increasing use of oral contraceptives also puts women at higher risk of thrombophilia than men. 1 10 Although the elderly formed the majority of patients on warfarin, a combined figure of all the younger patients (under 40 years) was highest. It can also be argued that age tends to limit mobility in the elderly, thereby putting them at higher risk of thrombophilia. 11

Questionnaire responses revealed that the majority (61.7%) of those on warfarin indicated that they were not aware of the need for regular check-ups, with 38% of the participants having missed their warfarin medication at least once and 25% taking warfarin overdose. These are some of the challenges contributing to the lack of adherence to medications. Due to these circumstances, warfarin monitoring and disease management in Zimbabwe becomes a big challenge. 12 13

Thirty-four percent of the participants admitted to taking alcohol while 23.4% indicated that they did not quit smoking after commencing warfarin therapy. Alcohol intake and smoking are known to increase and inhibit the effects of warfarin, respectively. While alcohol intake is linked to increased warfarin metabolism, smoking is known to be associated with increased warfarin dosage requirements. 1 8 14 Therefore, patients who admitted to indulging in these habits were increasing their risk of bleeding or thrombosis, respectively.

An assessment of the effectiveness of warfarin administration showed that a high number of patients were neither monitored for any concomitant contraindicated drugs nor advised on proper noncontraindicated diet. For example, foods with high vitamin K content (such as the Zimbabwean green vegetables: *bowora* , *tsunga* , and spinach) are known to reverse the effects of warfarin. Therefore, monitoring of warfarin treatment and maintaining the recommended diet are paramount in the effective drug action. Most of the patients on warfarin had monthly appointment dates, although it is recommended that patients on warfarin should be observed by their doctor or nurse after every 3 days following a 10-mg daily dose unless they are computer managed. 15

DVT was the main indication for warfarin therapy of all thrombophilia events, with highest prevalence among young adults and the elderly. The risk of venous thrombosis (comprising DVT) is known to increase sharply above the age of 45. This could explain why the majority of patients in the present study were in this age group. The major outcomes of venous thrombosis are death, postthrombotic syndrome, and excessive bleeding. 16 Therefore, it is particularly important to monitor warfarin therapy to avoid these outcomes.

Fig. 2 shows the general trend of participants' first 3 monthly consecutive INR values. These results were used as a tool to assess the effects of warfarin currently being prescribed at Parirenyatwa Group of Hospitals via the use of patients' INRs. The results showed a significant relationship among the differences of all the three classes, namely INR1 (first visit), INR2 (second visit), and INR3 (third visit). There was a statistically significant difference between INR1 and INR2 with a *p* -value < 0.0001, and similarly there was also a statistically significant difference between INR2 and INR3 ( *p* < 0.001). This is a good indication as the median INR pattern generally decreases as treatment moved from INR1 to INR3 eventually falling into therapeutic range, with targets of 2.5 (2–3 target range). This shows a glimpse of hope that despite all the negative pointers to the system, the warfarin is benefiting some of the patients.

## Conclusion

It can be concluded that patients who followed their treatment schedules for warfarin responded to the study very well. More females were on warfarin therapy than males. Most patients did not know about the benefits of regular check-ups, with some even missing their appointments or prescribed medications, or taking overdose. The monitoring system of patients on warfarin therapy appeared to be inadequate, which calls for a review of the current practice and need for a centralized system. However, the INR results showed that, if effectively monitored, warfarin therapy can be beneficial to thrombophilic patients if successfully managed.

## Limitations

This study was limited by the small number of patients who volunteered to take part as well as the short duration of 4 months, which was just one season.

## Recommendations

- A follow-up study should be conducted using larger sample size and a longer period, taking into consideration seasonal variations in thrombophilia diseases.
- It is strongly recommended that stand-alone facility to deal with thrombophilia challenges in Zimbabwe be established.
- A national standard model of care for warfarin therapy services should be agreed upon. This will handle all challenges and requirements for optimal anticoagulation therapy in general and warfarin specifically.
- Patient education and information sheets for the patients and their physicians would need to be prepared and made available.
